# Time-course responses of circulating microRNAs to three resistance training protocols in healthy young men

**DOI:** 10.1038/s41598-017-02294-y

**Published:** 2017-05-19

**Authors:** Shufang Cui, Biao Sun, Xin Yin, Xia Guo, Dingming Chao, Chunni Zhang, Chen-Yu Zhang, Xi Chen, Jizheng Ma

**Affiliations:** 1 State Key Laboratory of Pharmaceutical Biotechnology, Collaborative Innovation Center of Chemistry for Life Sciences, Jiangsu Engineering Research Center for MicroRNA Biology and Biotechnology, NJU Advanced Institute for Life Sciences (NAILS), School of life sciences, Nanjing University, Nanjing, Jiangsu 210046 China; 2 Department of Exercise and Heath, Nanjing sports Institute, 8 Linggusi Road Nanjing, Jiangsu, 210014 China; 3 The Lab of Military Conditioning and Motor Function Assessment, the PLA University of Science and Technology, 60 Shuang Long Jie Road, Nanjing, Jiangsu 211101 China; 4 Department of Clinical Laboratory, Jinling Hospital, Nanjing University School of Medicine, Nanjing, Jiangsu 210002 China

## Abstract

Circulating microRNAs (c-miRNAs) in human plasma have been described as a potential marker of exercise. The present study investigated the effects of three acute resistance training (RT) protocols on the time-course changes of the c-miRNAs profiles in young males. The subjects (n = 45) were randomly divided into three groups: muscular strength endurance (SE), muscular hypertrophy (MH) and maximum strength (MS). Venous blood samples were obtained before exercise and immediately, 1 h and 24 h after each RT protocol to assess the following biological parameters: c-miRNAs, anabolic and catabolic hormones, inflammatory cytokines and muscle damage markers. The results revealed that the levels of two c-miRNAs (miR-208b and miR-532), six c-miRNAs (miR-133a, miR-133b, miR-206, miR-181a, miR-21 and miR-221) and two c-miRNAs (miR-133a and miR-133b) changed significantly in response to the SE, MH and MS protocols (*p* < 0.05), respectively. The nature and dynamic processes of the c-miRNAs response were likely influenced by the RT modality and intensity. Moreover, miR-532 was negatively correlated with insulin-like growth factor-1 and positively correlated with interleukin-10, whereas miR-133a was negatively correlated with cortisol and positively correlated with testosterone/cortisol. These findings suggest that these c-miRNAs may serve as markers for monitoring the RT responses.

## Introduction

Resistance training (RT) is an effective method of exercise for improving muscular fitness^1–3^. Manipulation of acute program variables to efficiently achieve a specific training outcome (such as muscular hypertrophy, strength and local muscular endurance), has been proposed and recommended by many studies^1–3^. The preservation of muscle mass is necessary to maintain mobility and quality of life for apparently healthy adults of all ages, athletes and deconditioned older or frail individuals^2^.

The acute response to RT is an inherently complex and multifaceted phenomenon, which is difficult to gauge owing to the multitudinous training variables that contribute to the training load. Despite the complexity associated with quantifying RT, monitoring such responses or chronic adaptations is essential so that the exercises that best address individual needs and training objectives can be performed. Several RT protocols appear to potentiate traditional biomarker responses and have been developed to enhance various aspects of the neuromuscular system^1–3^, such as anabolic and catabolic hormones (e.g., testosterone, cortisol and insulin-like growth factor-1)^4, 5^, circulating inflammatory cytokines (e.g., interleukin-6, interleukin-10 and C-reactive protein)^6, 7^ and muscle damage markers (e.g., creatine kinase)^6^. These parameters may contribute to monitoring the training responses or neuromuscular adaptations^4–7^, including identification of the recovery period, adaptation patterns, direction and probable changes of these markers following different resistance exercise sessions^8, 9^.

Recently, microRNAs (miRNAs), which are expressed in a development-, cell type- and tissue-specific mode, have been established as crucial regulators of multiple biological phenomena^10^. Exercise has been shown to be an underlying activator of gene expression. Additionally, miRNAs play a central role in the post-transcriptional regulation of gene expression for a broad range of biological responses to various modes of exercise^11^. Accordingly, circulating miRNAs (c-miRNA) levels appear to differ between endurance and strength athletes^12^. Time-dependent alterations in c-miRNAs have been observed over several days during recovery from a muscular hypertrophy training protocol^13^. Thus, c-miRNAs have been proposed as promising biomarkers of exercise capacity.

It is known that several RT protocols, which differ in the configuration of the acute program variables (e.g., muscle action, loading and volume, selection of exercise pattern and order, rest periods, and repetition velocity and frequency), cause different physiological responses^1, 3^. At present, there is no consensus regarding the optimal way to control such variables. Due to the stability of c-miRNAs in plasma or serum^14^, c-miRNAs may be used as new biomarkers for a comprehensive evaluation of the specific adaptations induced by different exercises. Accordingly, knowledge of the time-course changes of different c-miRNAs in response to strength exercise may provide more information for monitoring the physiological stress induced by RT.

Therefore, the purpose of the present study was to investigate the time-course acute responses of c-miRNAs to different RT protocols: strength endurance (SE), muscular hypertrophy (MH) and maximum strength (MS). We hypothesized that acute changes of c-miRNAs would differ in response to the three RT protocols. Furthermore, the aim of our study was to examine whether the alterations of c-miRNAs were correlated with conventional anabolic and catabolic hormones, inflammatory cytokines and muscle damage biomarkers to gauge their physiological roles.

## Results

### Subject characteristics

The characteristics and one repetition maximum (1RM) of the subjects are shown in Table 1. Statistical analysis revealed that there were no baseline differences in the subject characteristics between groups.

**Table 1:** Clinical characteristics of participants and absolute load (kilograms) used during the RT session.

	Age (year)	BMI (kg·m^−2^)	Heart rate (beats·min^−1^)	Bench press (kg)	Squat (kg)	Pull down (kg)	Overhead press (kg)	Standing dumbbell curl (kg)
SE	19.36 ± 0.14	21.30 ± 0.25	74.64 ± 0.89	50.71 ± 1.23	82.86 ± 2.51	82.86 ± 1.98	36.96 ± 1.07	14.20 ± 0.39
MH	19.72 ± 0.20	22.12 ± 0.31	72.66 ± 1.41	53.10 ± 2.01	79.83 ± 1.50	84.66 ± 1.95	36.55 ± 1.22	15.00 ± 0.48
MS	18.87 ± 0.12	21.90 ± 0.30	72.10 ± 1.76	50.83 ± 1.57	85.83 ± 2.34	84.67 ± 2.17	31.75 ± 0.68	14.82 ± 0.41

Values represent the mean ± SEM obtained from 15 subjects separately in each of the three groups.

### Circulating blood parameters in response to the three resistance training protocols

For each subject, blood samples were collected before exercise (Pre), immediately after exercise (0 h), after 1 h of recovery (1 h) and after 24 h of recovery (24 h) for the three resistance protocols. Exercise significantly increased the blood concentrations of lactate (LA) 0 h postexercise when compared to Pre, with a different range for the three RT protocols (SE: 1.2 ± 0.1 mmol·L^−1^ vs. 9.0 ± 0.5 mmol·L^−1^, *p* < 0.05; MH: 1.1 ± 0.1 mmol·L^−1^ vs. 10.6 ± 0.6 mmol·L^−1^, *p* < 0.05; MS: 1.1 ± 0.1 mmol·L^−1^ vs. 4.8 ± 0.4 mmol·L^−1^, *p* < 0.05), and all nearly decreased to the baseline level at 1 h postexercise.

**Figure 1:**
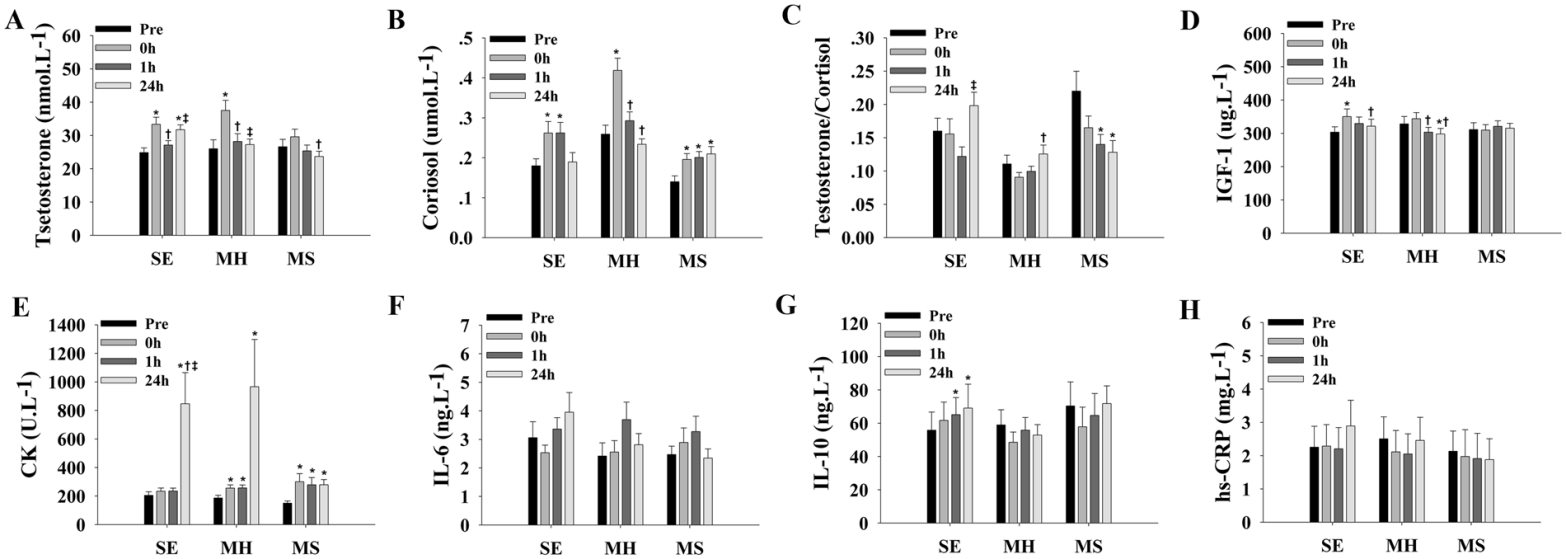
Alterations of blood parameters in response to three types of strength training protocol (n = 15 for each group). The plasma levels of hormones (**A,B,C and D**), muscle damage markers (**E**) and inflammatory cytokines (**F,G and H**) obtained at baseline (Pre), immediately after exercise (0 h), after 1 h of recovery (1 h) and after 24 h of recovery (24 h) for three resistance protocols (SE, MH, and MS). ^*^indicates differences compared to Pre, *p* < 0.05; ^†^indicates differences compared to 0 h, *p* < 0.05; ^‡^indicates differences compared to 1 h, *p* < 0.05. Values represent the mean ± SEM.

The data for hormones, muscle damage biomarkers and inflammatory cytokines in response to the three RT protocols are summarized in Fig. 1. Both SE and MH protocols could induce significant changes of plasma testosterone and cortisol levels, with peak values observed 0 h postexercise (*p* < 0.05) (Fig. 1A,B). Plasma levels of cortisol were increased in response to MS, peaking after 24 h recovery (*p* < 0.05) (Fig. 1B). All of the three protocols could change the ratios of testosterone and cortisol (T/C), peaking for SE and MH whereas declining to minimum for MS after 24 h recovery (*p* < 0.05) (Fig. 1C). The plasma levels of insulin-like growth factor-1 (IGF-1) were altered in response to SE and MH, with peak values observed 0 h postexercise (*p* < 0.05) for SE whereas minimum values observed after 24 h recovery (*p* < 0.05) for MS (Fig. 1D).

Plasma levels of creatine kinase (CK) were affected by the three protocols, with peak values observed after 24 h recovery (*p* < 0.05) for SE and MH, and 0 h postexercise (*p* < 0.05) for MS (Fig. 1E). The plasma levels of interleukin-6 (IL-6), interleukin-10 (IL-10) and hypersensitive C-reactive protein (hs-CRP) did not respond to MH and MS (*p* values between 0.07 and 0.98) (Fig. 1F–H). The plasma IL-10 levels were increased for SE, peaking after 24 h recovery (*p* < 0.05) (Fig. 1G).

### Circulating miRNAs screening by TaqMan Low Density Array

**Figure 2:**
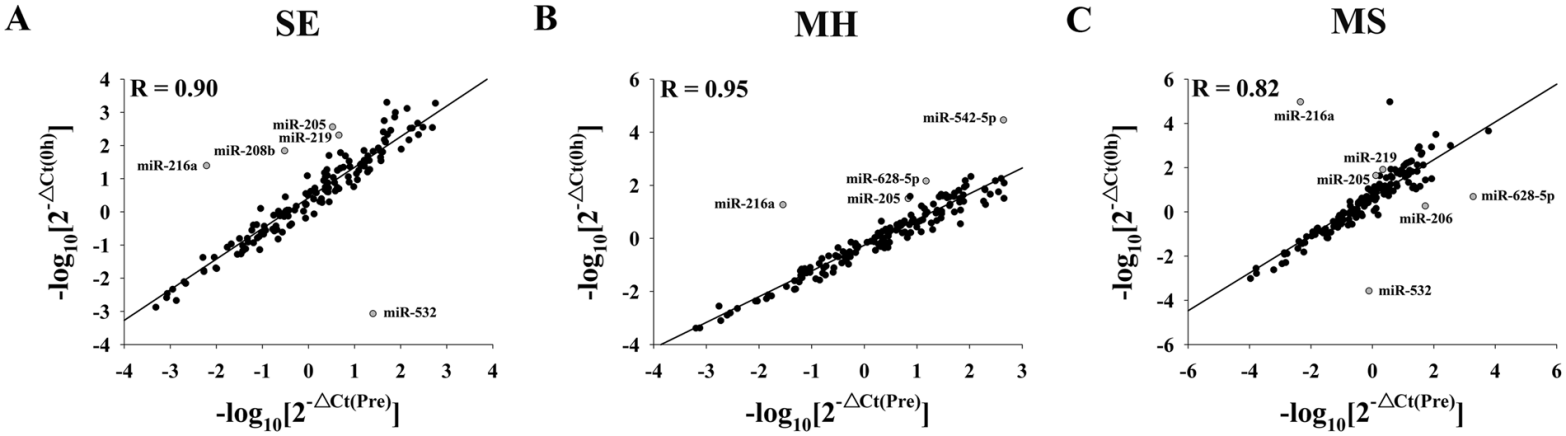
Pearson correlation scatter plot of the circulating miRNA levels for SE (**A**), MH (**B**) and MS (**C**) as determined by TLDA. The Ct values were normalized to the calculated mean Ct value of the Let-7d/g/i of each pooled sample (ΔCt).

The global plasma miRNAs expression patterns in response to the SE, MH and MS protocols were analyzed using TaqMan Low Density Array (TLDA). The Pearson correlation coefficient (R) for the three paired groups was 0.90 for SE, 0.95 for MH, and 0.82 for MS (Fig. 2A–C). A miRNA was considered to be differently changed if its Ct value was below 35 and if there was a larger than 2-fold change in concentration. Class-comparison analysis of all 754 human miRNAs showed that plasma miRNAs responded differently to the three RT protocols (SE: 1 miRNA increased, 93 miRNAs decreased; MH: 75 miRNAs increased, 7 miRNAs decreased; MS: 16 miRNAs increased, 60 miRNAs decreased). The markedly altered miRNAs in plasma (fold change > 10) after the SE, MH, and MS protocols are listed in Table 2. As shown in Fig. 2, 8 miRNAs (miR-205, miR-206, miR-208b, miR-216a, miR-219, miR-532, miR-542-5p and miR-628-5p) were considered for subsequent validation individually because they showed the greatest fold change in response to (1) one of the three RT protocols only (miR-208b for SE, miR-542-5p for MH, and miR-206 for MS), (2) two of the three RT protocols (miR-219 and miR-532 for SE and MS, and miR-628-5p for MH and MS), or (3) all of the three RT protocols (miR-216a and miR-205). Additionally, given that exercise can induce disturbances in skeletal muscle structure and function as well as a cascade of inflammatory responses^9^, 8 miRNAs related to muscle or inflammation were also selected, including miR-1, miR-133a, miR-133b, miR-146a, miR-181a, miR-21, miR-221 and miR-378^15–17^. Five of the selected miRNAs (miR-1, miR-133a, miR-133b, miR-206 and miR-208b) belong to the myomiRs, which are exclusively or preferentially expressed in muscle^18^. All of the 16 selected miRNAs were quantified individually among the three RT protocols.

**Table 2:** Markedly altered miRNAs in pooled plasma samples from baseline (Pre) compared with those from immediately after exercise (0 h) in the SE, MH and MS groups as determined by TLDA.

SE				MH				MS			
	Relative expression		Log_2_(Fold change)		Relative expression		Log_2_(Fold change)		Relative expression		Log_2_(Fold change)
miRNA	Pre	0 h		miRNA	Pre	0 h		miRNA	Pre	0 h	
**Increased**				**Increased**				**Increased**			
miR-532-5p	0.04001	1168.79638	14.83	miR-214–3p	0.01499	0.28228	4.24	miR-532–5p	1.30000	12242.07083	13.20
**Decreased**				miR-551b-5p	0.00223	0.03075	3.79	miR-628–5p	0.00053	0.66777	10.31
miR-184	0.20166	0.01648	−3.61	miR-31–5p	0.00499	0.05294	3.41	miR-206	0.01914	1.77427	6.53
miR-148b-3p	0.02277	0.00178	−3.68	**Decreased**				**Decreased**			
miR-135b-5p	0.01304	0.00100	−3.70	miR-628–5p	0.06715	0.00685	−3.29	miR-205–5p	0.77325	0.07342	−3.40
miR-320b	1.06079	0.08151	−3.70	miR-542–5p	0.00227	0.00003	−6.05	miR-219–5p	0.46054	0.03957	−3.54
miR-99b-3p	10.93159	0.78567	−3.80	miR-216a	35.10579	0.05364	−8.97	miR-204–5p	0.27257	0.00003	−13.29
miR-551b-5p	0.36030	0.01997	−4.17					miR-216a	222.05428	0.00003	−22.62
miR-183–5p	0.02004	0.00050	−5.33								
miR-219–5p	0.21909	0.00485	−5.50								
miR-205–5p	0.30140	0.00275	−6.78								
miR-208b	3.30777	0.01446	−7.84								
miR-216a	163.23403	0.04021	−12.29								

The relative expression was expressed based on the formula (2^−△Ct^) and the Ct values were normalized on the calculated mean Ct value of the Let-7d/g/i of each pooled sample (ΔCt). Fold changes of miRNAs species were calculated using the 2^−ΔΔCt^ method.

### Circulating miRNAs in response to the acute muscle endurance protocol and association with conventional biomarkers

For each of the three groups, the Ct values for Let-7d/g/i before and after resistance exercise showed low variability (see Supplementary Fig. S1). The plasma levels of four myomiRs (miR-1, miR-133a, miR-133b and miR-206) and other tissue-specific miRNAs or unknown origin miRNAs displayed no significant changes at different time points (*p* values between 0.06 and 0.97). The relative concentrations of the 16 c-miRNAs are presented in Table 3.

**Table 3:** The relative concentrations of 16 plasma miRNAs chosen from TLDA determined by RT-qPCR assay for the SE, MH and MS protocols.

miRNA	SE				MH				MS			
	Pre	0 h	1 h	24 h	Pre	0 h	1 h	24 h	Pre	0 h	1 h	24 h
miR-1	0.14 ± 0.02	0.17 ± 0.05	0.22 ± 0.04	0.12 ± 0.03	0.24 ± 0.05	0.19 ± 0.05	0.23 ± 0.05	0.33 ± 0.13	0.27 ± 0.06	0.24 ± 0.04	0.31 ± 0.06	0.29 ± 0.06
miR-133a	0.46 ± 0.09	0.46 ± 0.17	0.46 ± 0.11	0.43 ± 0.15	0.33 ± 0.04	0.17 ± 0.55*	0.33 ± 0.06	0.28 ± 0.04	1.34 ± 0.30	0.90 ± 0.19*	1.22 ± 0.27	1.17 ± 0.36
miR-133b	0.11 ± 0.02	0.12 ± 0.02	0.14 ± 0.03	0.11 ± 0.01	0.10 ± 0.01	0.08 ± 0.01	0.12 ± 0.04	0.14 ± 0.02^†^	0.13 ± 0.02	0.11 ± 0.01	0.14 ± 0.02^†^	0.11 ± 0.01
miR-146a	4.01 ± 0.21	3.65 ± 0.10	6.23 ± 2.01)	4.17 ± 1.07	1.63 ± 0.38	1.15 ± 0.25	1.01 ± 0.21	1.26 ± 0.22	1.99 ± 0.31	2.33 ± 0.09	2.15 ± 0.10	1.79 ± 0.18
miR-181a	0.1 ± 0.02	0.08 ± 0.02	0.09 ± 0.01	0.07 ± 0.01	0.07 ± 0.02	0.07 ± 0.02	0.12 ± 0.03*^,†^	0.06 ± 0.01^‡^	0.07 ± 0.01	0.06 ± 0.01	0.07 ± 0.01	0.05 ± 0.01
miR-21	5.87 ± 0.67	4.35 ± 1.28	6.77 ± 0.99	4.89 ± 0.75	5.66 ± 0.79	3.93 ± 0.71*	6.62 ± 1.00^†^	5.17 ± 1.10	3.37 ± 0.63	3.03 ± 0.53	3.16 ± 0.52	2.63 ± 0.37
miR-205	0.08 ± 0.01	0.06 ± 0.01	0.08 ± 0.01	0.08 ± 0.02	0.10 ± 0.01	0.12 ± 0.05	0.10 ± 0.01	0.10 ± 0.01	0.13 ± 0.02	0.13 ± 0.01	0.15 ± 0.02	0.14 ± 0.02
miR-206	14.6 ± 2.32	13.1 ± 2.63	16.9 ± 3.23	13.1 ± 3.31	11.6 ± 1.60	8.9 ± 0.96	14.0 ± 1.34	10.7 ± 0.99^†^	10.4 ± 1.61	8.2 ± 1.35	11.3 ± 1.90	7.3 ± 1.55
miR-208b	0.22 ± 0.04	0.13 ± 0.03*	0.17 ± 0.03	0.12 ± 0.03*	0.23 ± 0.06	0.17 ± 0.02	0.22 ± 0.07	0.24 ± 0.08	0.23 ± 0.03	0.17 ± 0.03	0.14 ± 0.02	0.16 ± 0.03
**miR-216a**	**ND**				**ND**				**ND**			
miR-219	0.06 ± 0.01	0.05 ± 0.01	0.05 ± 0.01	0.04 ± 0.01	0.07 ± 0.01	0.06 ± 0.01	0.06 ± 0.01	0.07 ± 0.01	0.04 ± 0.01	0.05 (0.01)	0.06 ± 0.01	0.06 ± 0.01
miR-221	0.27 ± 0.02	0.31 ± 0.04	0.29 ± 0.03	0.24 ± 0.01	0.13 ± 0.01	0.16 ± 0.02	0.08 ± 0.69^†^	0.15 ± 0.02^‡^	0.15 ± 0.07	0.12 ± 0.04	0.12 ± 0.05	0.12 ± 0.01
miR-378	2.84 ± 0.30	2.48 ± 0.31	2.85 ± 0.49	2.54 ± 0.17	3.34 ± 0.41	4.08 ± 0.72	3.90 ± 0.56	3.73 ± 0.50	3.20 ± 1.03	3.05 ± 0.1	3.25 ± 0.45	2.98 ± 0.56
miR-532	0.53 ± 0.08	0.61 ± 0.08	0.64 ± 0.08*	0.69 ± 0.08*	0.85 ± 0.57	0.77 ± 0.47	0.57 ± 0.28	0.86 ± 0.57	0.69 ± 0.05	0.58 ± 0.06	0.53 ± 0.08	0.50 ± 0.09
miR-542	0.04 ± 0.01	0.03 ± 0.01	0.03 ± 0.01	0.03 ± 0.01	0.07 ± 0.01	0.06 ± 0.01	0.07 ± 0.01	0.06 ± 0.01	0.03 ± 0.01	0.03 ± 0.01	0.03 ± 0.01	0.03 ± 0.01
miR-628	0.42 ± 0.03	0.39 ± 0.02	0.39 ± 0.04	0.34 ± 0.03	0.33 ± 0.06	0.34 ± 0.06	0.32 ± 0.05	0.29 ± 0.05	0.47 ± 0.02	0.42 ± 0.02	0.49 ± 0.04	0.41 ± 0.02

The relative concentrations of the circulating miRNAs were normalized to the Let-7d/g/i and presented as the mean ± SEM. miRNAs were obtained from plasma samples at baseline (Pre), immediately after exercise (0 h), after 1 h of recovery (1 h), after 24 h of recovery (24 h). *p < 0.05 compared to Pre; ^†^ *p* < 0.05 com*p*ared to 0 h; ^‡^ *p* < 0.05 compared to 1 h. ND, non-detected values. Values obtained from 15 subjects separately in each of the three groups.

**Figure 3:**
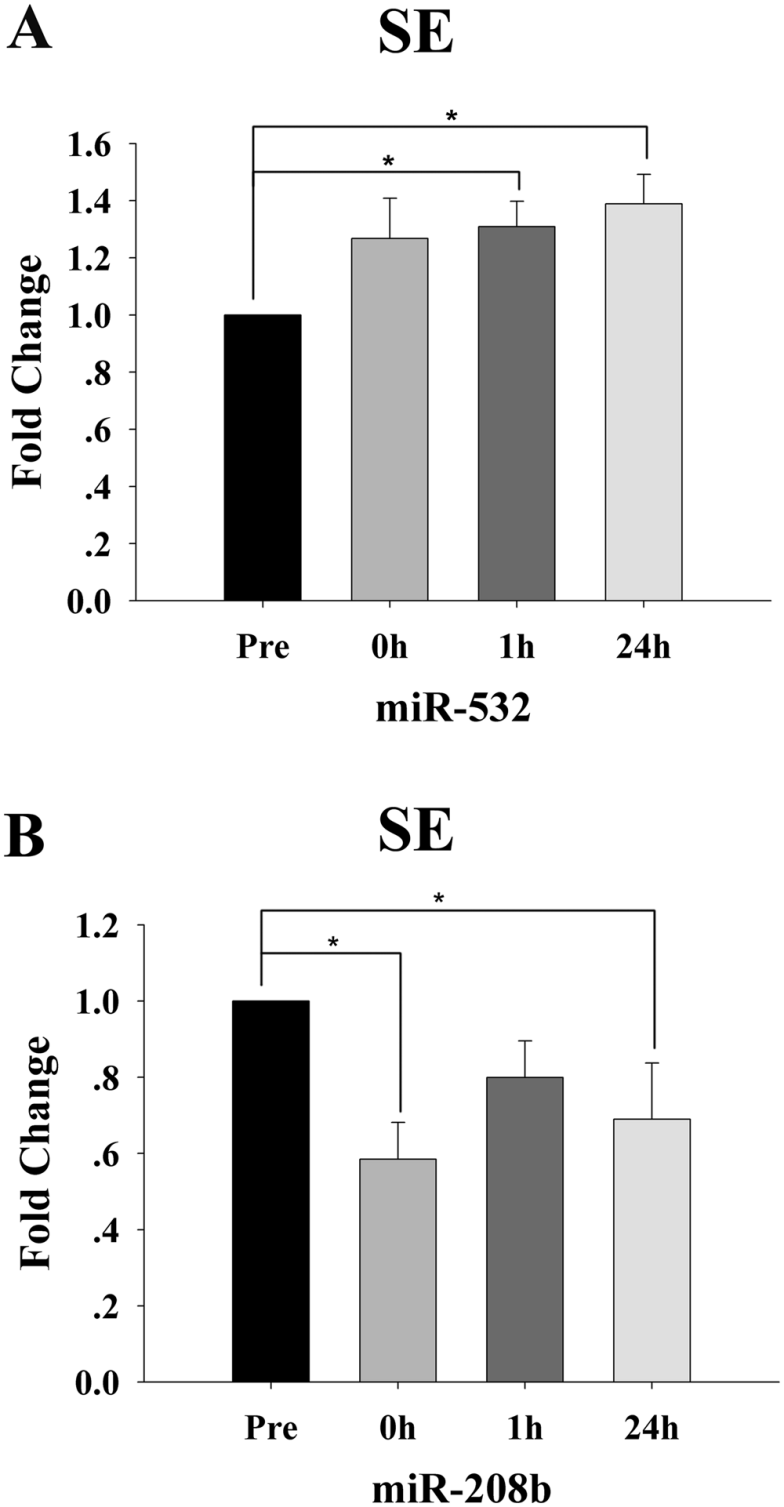
Time-course changes in the plasma levels of miR-532 (**A**) and miR-208b (**B**) for the resistance endurance (SE) protocol (n = 15). For each subject, the c-miRNA levels at baseline (Pre) were assigned a fold change of 1, and measurements obtained immediately after exercise (0 h), after 1 h of recovery (1 h) and after 24 h of recovery (24 h) were compared to the baseline. *Indicates differences compared to Pre, *p* < 0.05. Values represent the mean ± SEM.

However, the plasma levels of miR-532 were markedly increased after 1 h recovery (*p* < 0.05) and remained elevated after 24 h recovery (*p* < 0.05) (Fig. 3A). Additionally, the plasma levels of another myomiR, miR-208b, were observably decreased 0 h postexercise (*p* < 0.05) and did not return to the basal level after 24 h recovery (*p* < 0.05) (Fig. 3B).

**Figure 4:**
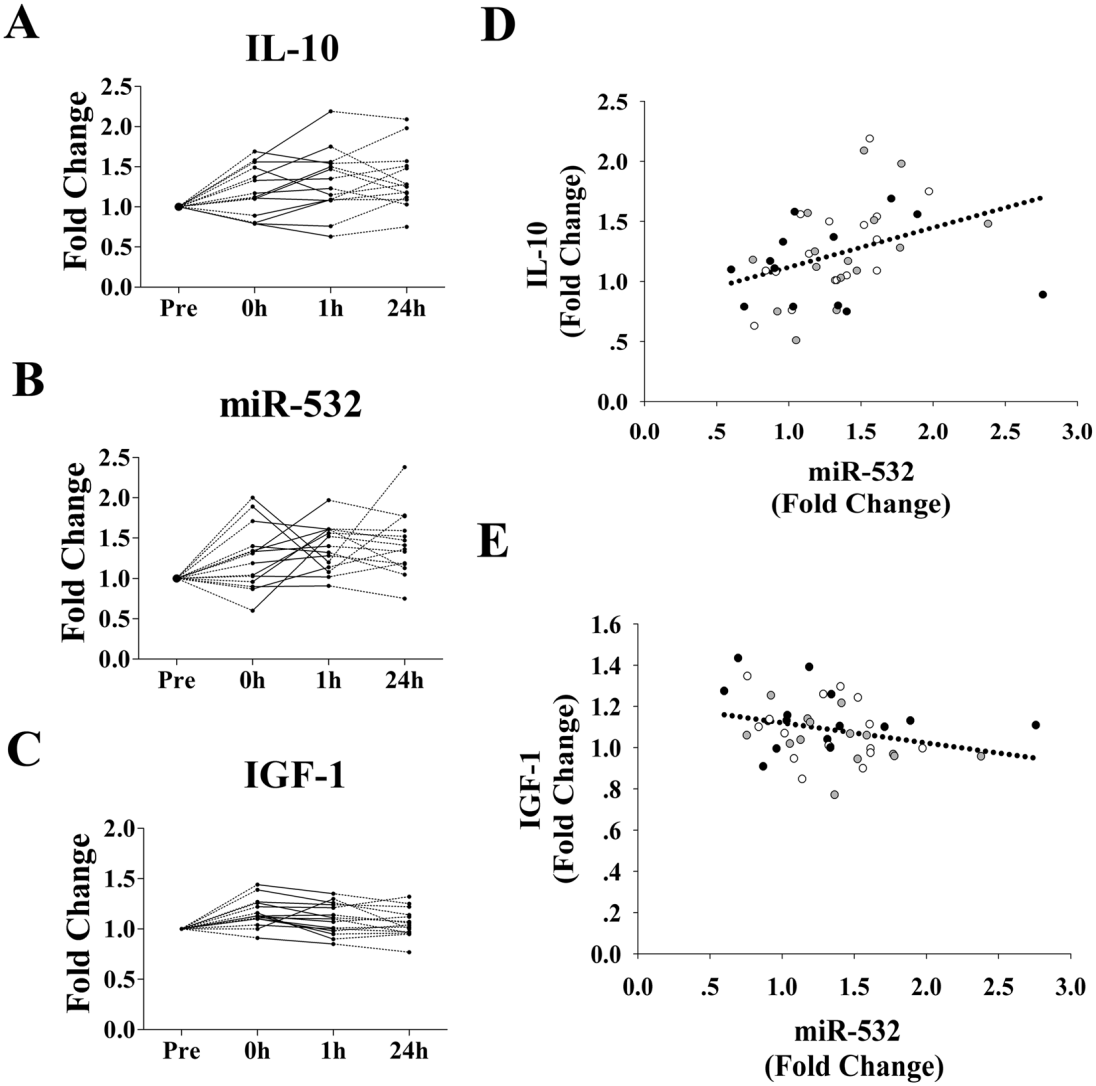
Alterations in miR-532 are directly correlated with changes in plasma IL-10 and IGF-1 levels (n = 15). For each subject, the plasma levels of miR-532, IL-10 and IGF-1 at baseline (Pre) were assigned a fold change of 1, and measurements obtained immediately after exercise (0 h), after 1 h of recovery (1 h) and after 24 h of recovery (24 h) were compared to the baseline. Scatterplots show the plasma levels of IL-10 (**A**), miR-532 (**B**) and IGF-1 (**C**). A direction correlation is observed between the levels of plasma miR-532 and IL-10 (**D**) and IGF-1 (**E**). The correlation analysis indicates the sum result of the three time points (black dots indicate the values for 0 h; white dots indicate the values for 1 h; and gray dots indicate the values for 24 h).

In particular, IL-10 displayed a consistent increase (Fig. 4A) similar to that for miR-532 (Fig. 4B). A distinct positive correlation (R = 0.42, *p* = 0.004; Fig. 4D) was observed between changes in plasma levels of miR-532 and IL-10. Moreover, a significant negative correlation (R = −0.33, *p* = 0.03) was observed between alterations in the plasma miR-532 level and the IGF-1 level (Fig. 4B,C,E).

### Circulating miRNAs in response to the acute muscle hypertrophy protocol and association with conventional biomarkers

**Figure 5:**
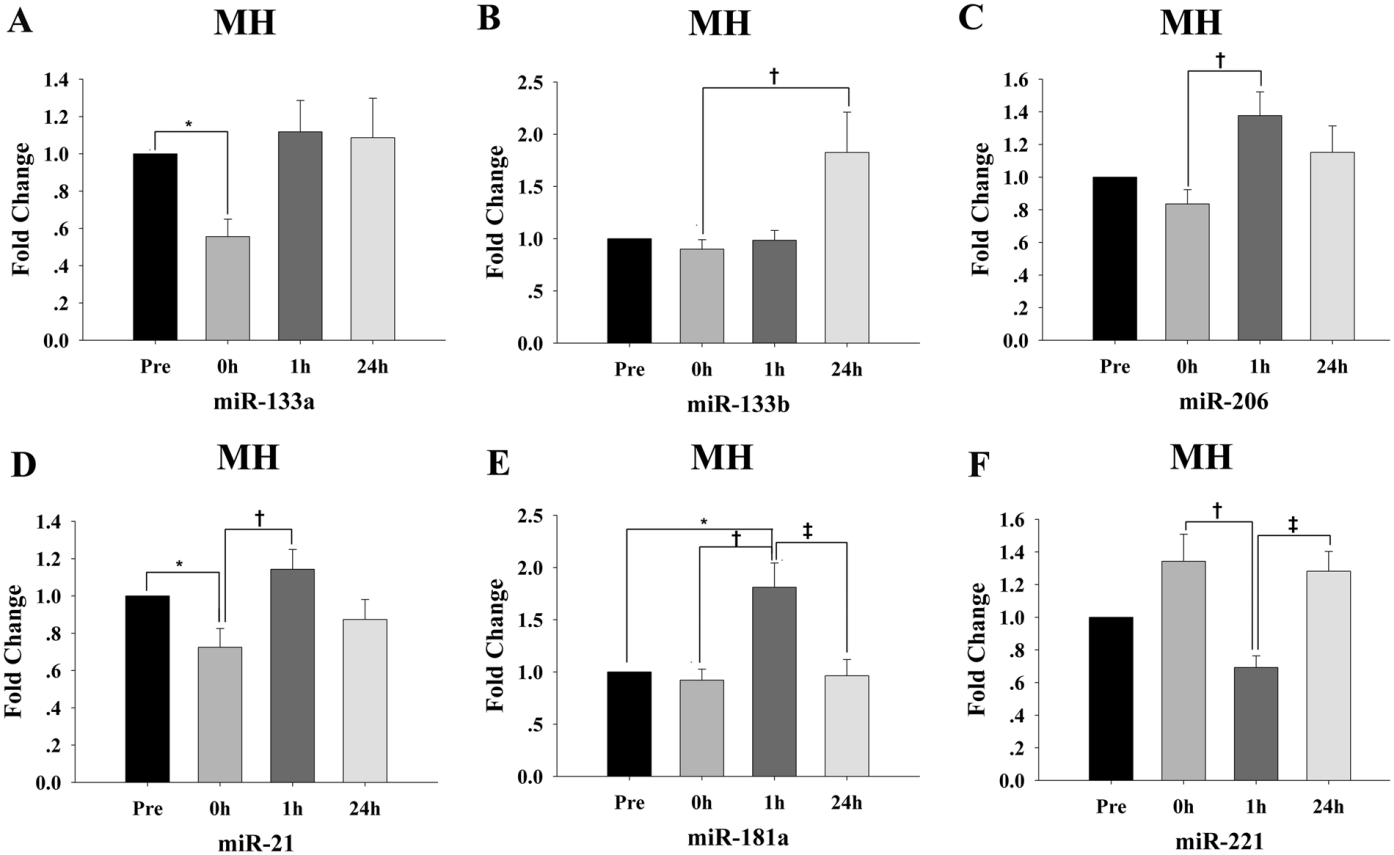
Time-course changes in specific circulating miRNAs for the muscle hypertrophy (MH) protocol (n = 15). (**A–F**) For each participant, the c-miRNA levels at baseline (Pre) were assigned a fold change of 1, and measurements obtained immediately after exercise (0 h), after 1 h of recovery (1 h) and after 24 h of recovery (24 h) were compared to the baseline. ^*^indicates differences compared to Pre, *p* < 0.05; ^†^indicates differences compared to 0 h, *p* < 0.05; ^‡^indicates differences compared to 1 h, *p* < 0.05. Values represent the mean ± SEM.

Three myomiRs, miR-133a, miR-133b and miR-206 that were not affected by SE, showed different expression patterns in response to MH. The plasma levels of miR-133a were significantly decreased (*p* < 0.05) 0 h postexercise, and restored to the baseline level after 1 h recovery (*p* > 0.05) (Fig. 5A). The plasma levels of miR-133b level were markedly increased after 24 h recovery (*p* < 0.05) (Fig. 5B). The plasma miR-206 reached peak levels after 1 h recovery (*p* < 0.05) and decreased to the baseline level after 24 h recovery (*p* > 0.05) (Fig. 5C).

Additionally, other muscle or inflammation-related miRNAs also responded to MH. The plasma levels of miR-21 were markedly decreased 0 h postexercise (*p* < 0.05) and reached peak values after 1 h recovery (Fig. 5D). The plasma miR-181a levels peaked after1 h recovery (*p* < 0.05) and decreased to the baseline value after 24 h recovery (*p* > 0.05) (Fig. 5E). The plasma levels of miR-221 displayed a nonsignificant increase 0 h postexercise, following with a significant decrease after 1 h recovery (*p* < 0.05) and increased again after 24 h recovery (*p* < 0.05) (Fig. 5F).

No distinct changes in the plasma levels of the other 10 miRNAs were observed (*p* values between 0.06 and 0.96) (Table 3). There were no correlations between the levels of the six changed c-miRNAs and conventional parameters (R values between −0.43 and 0.43, *p* values between 0.08 and 0.96).

### Circulating miRNAs in response to the maximum muscle resistance protocol and association with conventional biomarkers

**Figure 6:**
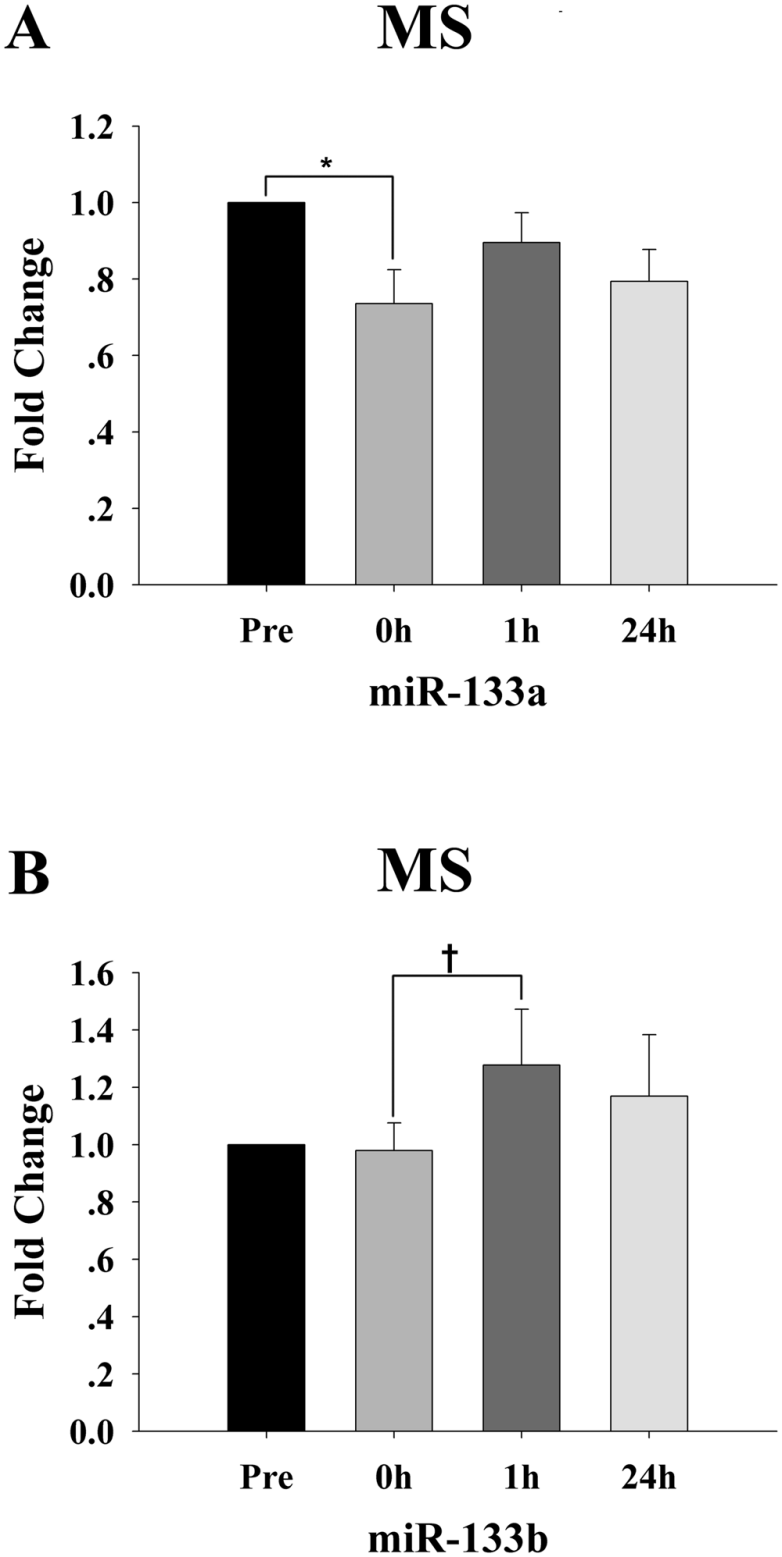
Time-course changes in the plasma levels of miR-133a (**A**) and miR-133b (**B**) for the maximum strength (MS) protocol (n = 15). For each subject, the c-miRNA levels at baseline (Pre) were assigned a fold change of 1, and measurements obtained immediately after exercise (0 h), after 1 h of recovery (1 h) and after 24 h of recovery (24 h) were compared to the baseline. ^*^Indicates differences compared to Pre, *p* < 0.05; ^†^indicates differences compared to 0 h, *p* < 0.05. Values represent the mean ± SEM.

Two myomiRs, miR-133a and miR-133b which responded to MH, were also affected by MS. The plasma levels of miR-133a reached its minimum 0 h postexercise (*p* < 0.05) and restored to the basal level after 1 h recovery (*p* > 0.05) (Fig. 6A). The plasma miR-133b levels were not observably changed 0 h postexercise, peaking after 1 h recovery (*p* < 0.05) (Fig. 6B).

**Figure 7:**
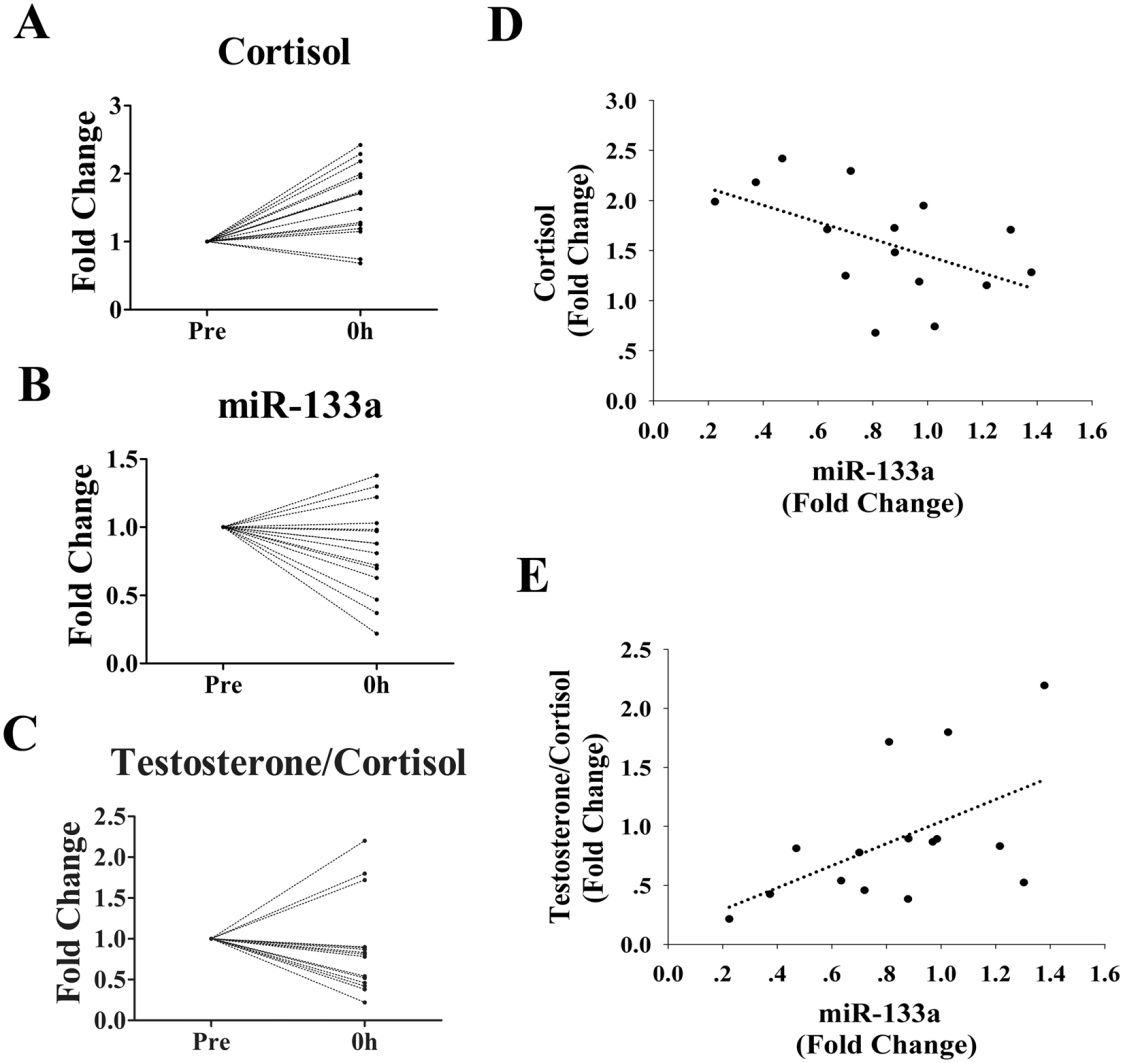
Correlations of miR-133a with plasma cortisol and testosterone/cortisol levels (n = 15). For each subject, the plasma levels of miR-133a, cortisol and testosterone at baseline (Pre) were assigned a fold change of 1, and measurements obtained immediately after exercise (0 h), after 1 h of recovery (1 h) and after 24 h of recovery (24 h) were compared to the baseline. Scatterplots show the plasma levels of cortisol (**A**), miR-133a (**B**) and testosterone/cortisol (**C**). A direct correlation is observed between plasma levels of miR-133a and cortisol (**D**) and testosterone/cortisol (**E**).

Additionally, no significant changes were found for other c-miRNAs at different time points (*p* values between 0.13 and 0.97) (Table 3). Correlation analysis indicated that changes in miR-133a had a negative correlation with cortisol (R = −0.53, *p* = 0.04; Fig. 7A,B,D) and a positive correlation with T/C (R = 0.59, *p* = 0.02; Fig. 7B,C,E).

### Summary of the validated and predicted targets of the changed circulating miRNAs

Subsequently, we summarized the validated target genes regulated by the altered plasma miRNAs which responded to the three RT protocols. The target genes and their functions that involved in muscle biogenesis and structure as well as exercise induced adaptations (such as inflammations and angiogenesis) are shown as Supplementary Table S1. To explore the potential roles of emerging miR-532, we performed a bioinformatics prediction of the possible targets via the use of multiple target prediction databases (DIANA, Microinspector, Miranda, Mirtarget, Mitarget, Nbmirtar, Pictar, Pita, Rna22, Rnahybrid and Targetscan). The targets which were simultaneously predicted by at least five prediction databases were selected for subsequent Gene Ontology (GO) analysis. The predicted miR-532 targets and the involved biological processes which are potentially related to exercise induced adaptations (such as energy metabolism and immune response) are listed in Table 4.

**Table 4:** The predicted targets of miR-532 and the involved biological processes analyzed by Gene Ontology.

Biological process	Predicted targets
Generation of precursor metabolites and energy (GO: 0006091)	CS
	PYROXD1
	ERO1L
Carbohydrate metabolic process (GO: 0005975)	DBT
	NMUR2
	CS
	ARSJ
	PPP1R3A
	SI
	ARSG
	CDK6
	PPP1R3D
Lipid metabolic process (GO: 0006629)	B3GNT1
	NR2C2
Tricarboxylic acid cycle (GO: 0006099)	CS
Immune response (GO: 0006955)	ULBP1
	IL6ST
	NCF2
	NFKB1
	TNFRSF1B
	GBP1
	SESTD1

## Discussion

Each RT modality leads to differentiated molecular and structural adaptations not only in the exercised muscle but also in distant tissues, resulting in a whole body adaptive response^1^. Overall, the results of the present study suggest that RT can lead to distinct time course changes in the profiles of c-miRNAs, and these changes likely depend on the RT modality or intensity. Moreover, the correlations of miR-532 and miR-133a in particular with conventional parameters suggested their potential roles as biomarkers of resistance exercise.

Regimented RT is well-established as an effective mechanism to achieve a specific training outcome by specifying acute RT program variables^1^. Although these training variables are conducive to good performance and post-exercise adaptations to resistance exercise, the relative intensity (%1RM) appears to be a key factor^19^. Additionally, maximizing the specific response to RT is thought to be best achieved by proper manipulation of exercise^1–3^. Specific signaling pathways are critical for the structural remodeling and functional adjustment of skeletal muscle in response to exercise-induced physiological and biochemical stimuli^20, 21^. The principle of super-compensation is necessary to increase exercise stimulus and for adaptations to occur^22^. The time-course changes for an acute hormonal and cytokine or c-miRNA response to RT might provide a thorough understanding of the molecular events that occur during exercise and later recovery phases.

Traditionally, changes in anabolic-catabolic hormones, muscle damage markers and inflammatory mediators have been widely used to monitor and understand these acute program variables^23^. In our study, these conventional biomarkers, such as testosterone, cortisol, T/C, IGF-1, CK and IL-10 showed different dynamic responses to the different RT protocols. While the pro-inflammatory biomarkers, such as IL-6 and hs-CRP^24^, did not show any changes at different time points. These results are in partly consistent with previous studies^24, 25^. At present, although the mechanism about exercise induced release of these biomarkers remains incompletely understood, the physiological implications have been addressed^24, 25^.

The c-miRNAs, which are present and highly stable in the bloodstream, have been proposed as potential new biomarkers of specific exercise responses^12, 15, 26, 27^. In our study, the plasma levels of several myomiRs (including miR-133a, miR-133b, miR-206 and miR-208b) showed dynamic changes in response to RT, whereas the plasma miR-1 did not respond to RT. Such differential changes for these myomiRs in circulation might indicate the different degrees of muscle fiber recruitment or stress/adaptations in response to RT. Additionally, the direct correlations between the changes in plasma cortisol level and the T/C ratio and miR-133a level that were observed for the MS protocol likely indicated stressful training (overreaching)^1^. Moreover, cortisol was significantly, albeit weakly, associated with gains in type II fiber area^28^. Thus, the plasma miR-133a level may be a potentially useful biomarker of an actual physiological strain or tissue-remodeling processes for the MS protocol.

In the present study, other c-miRNAs besides myomiRs also displayed different responses to the three RT protocols. For the SE protocol, time-course analysis showed that the plasma miR-532 level increased during early recovery and remained elevated for a long time. For the MH protocol, the plasma levels of three c-miRNAs, miR-21, miR-181a and miR-221, also showed dynamic changes. The plasma levels of miR-21, which plays a crucial role in the inflammatory response^29^, has been found to change after a single bout of endurance exercise^15^. In our study, the time course of the plasma miR-21 level, starting with a decrease immediately after exercise followed by an increase during early recovery, may reflect the balance between the initial pro-inflammatory and later immunoregulatory anti-inflammatory responses^29^. There is increasing evidence that many of the pro-oxidative and pro-inflammatory processes that occur after acute exercise may be vital for the long-term adaptive responses to exercise training^30^. The plasma levels of miR-181a, a possible biomarker of acute muscle wasting^31^, was increased during early recovery. Moreover, the plasma miR-221 which is related to vascular biology and fat and glucose metabolism^32^, also showed dynamic responses in the present study. Additionally, the plasma miR-146a level, which increased in response to acute endurance exercise^26^ and decreased 3 days post-resistance exercise^13^, did not respond to RT in our study. The plasma levels of miR-378 which is associated with muscle mass gains *in vivo* ^33^, remained unchanged for the three RT protocols. The differences between the findings of our study and other studies may relate to exercise modality and the timing of the blood draws or training state^12, 26, 33^.

Furthermore, a negative correlation between the changes in plasma levels of IGF-1 and miR-532, as well as a positive correlation between that of IL-10 and miR-532 were observed in the present study. The increased IGF-1 level may exert positive effects on adaption to resistance exercise, i.e., muscle hypertrophy or connective tissue^25^. Moreover, exercise training improved the inflammatory profile by increasing the levels of the anti-inflammatory cytokine IL-10 in post-myocardial infarction patients^34^. In consideration of the correlations being the combined results for all data points, it is conceivable that there were c-miRNA-hormone stress or adaptation associations. Moreover, the predicted target genes of miR-532 revealed the potential role of miR-532 in metabolism processes or inflammation response related to RT. Thus, miR-532 may also be a biomarker for the beneficial effect of physical exercise or tissue-remodeling processes in response to RT.

Taken together, these results suggest that not all c-miRNAs from skeletal muscle or other tissues respond to an RT stimulus. Furthermore, the variation of c-miRNAs and traditional blood parameters noted above is likely related to the type or amount of muscle activated during resistance exercise. In our study, the absolute workloads for the three RT protocols were the same, thus the differential expression of c-miRNAs may be related to relative intensity. Plasma miRNAs, such as miR-133a, miR-133b, miR-181a, miR-206, miR-208b, miR-21, miR-221 and miR-532, may therefore represent important novel indicators of skeletal muscle, inflammation or metabolism stress or adaptations to RT protocols with different intensity.

Currently, whether the RT induced dynamic changes in c-miRNAs during exercise and the recovery phase directly reflect intracellular miRNA turnover is unknown. A previous study showed that extracellular dystrophy-associated miRNA levels show a dynamic mode of expression that mirrors the process of muscle pathology^35^. It is likely that the levels of different c-miRNAs induced by RT protocols may reflect specific training-related dynamic activities. Thus, c-miRNAs could be useful biomarkers for physiological mediators of exercise-induced stress or adaptations during exercise or the recovery phase.

The mechanism and function underlying the uptake and release of c-miRNAs following exercise are still unclear^36, 37^, and need to be studied further. However, characterizing these responses is an important step in understanding of the roles of exercise induced c-miRNA changes. Exercise induced c-miRNA elevations may represent a more generalized response to internal and/or external stress. Factors, including mechanical, oxidative or nitrosative stress, damaged cells^26^, changes in blood cell numbers^38^ or hemolysis^39^ and the release of secreted extracellular vesicles (exosomes)^40^, likely induce c-miRNAs production during exercise. However, c-miRNAs responded differently to the RT protocols in the present study, indicating a specific response to the demands of the exercise rather than a global exercise-induced c-miRNA response.

In summary, the findings of the present study indicate that RT can lead to changes in c-miRNA levels with transient and delayed kinetics. The amounts and differential expression of c-miRNAs in response to acute RT, which potentially depend on the relative intensity or other RT variables, may point to a physiological role in the phenotypic changes and metabolic and inflammatory processes induced by exercise.

## Limitations

This study has some limitations. First, the post-exercise analysis was only limited to 24 h after exercise, thus the delayed kinetics changes in c-miRNA profiles for a longer period of recovery were undetected. Second, the participants in the present study had maintained a regular exercise regimen for a period of time. The RT-associated alterations in some c-miRNAs are likely distinct between the untrained state and trained state. Additionally, we only analyzed the differences of the c-miRNAs and blood parameters in the same RT protocol. For practical and ethical reasons, the three RT protocols were performed by different individuals. To avoid the statistical difference that may be caused by the intrinsic and inherent discrepancy of different participants, we did not compare the c-miRNAs and conventional parameters among different RT protocols.

## Materials and Methods

### Subjects

Forty-five university cadets who led similar lives were requested to volunteer for this study. The participants had maintained a regular exercise regimen for 10 months, and none had weight machine training experience. Exclusion criteria included any history of neuromuscular, cardiovascular, hormonal and metabolic diseases. The subjects were prohibited from taking any medications, and the subjects maintained the same dietary intake throughout the study.

Blood samples were collected according to protocols approved by the Human Research Ethics Committee of Nanjing University. Written informed consent was obtained from all of the subjects. The Human Research Ethics Committee of Nanjing University approved the study protocol in conformity with the Declaration of Helsinki, and all experiments were carried out in accordance with approved guidelines of the Nanjing University.

### Experimental design

The forty-five students were randomly assigned to one of three groups: strength endurance exercise group (SE), muscular hypertrophy group (MH) and maximum strength group (MS). One week was dedicated exclusively to the individual’s 1RM test and familiarization of the participants with the equipment and exercise protocol. For each resistance exercise protocol, blood was collected before (Pre), immediately after exercise (0 h), 1 h after exercise (1 h) and 24 h after exercise (24 h) to assess the c-miRNAs and traditional biomarkers. For each group, the blood samples drawn before the exercise were set as a control.

### Resistance exercise protocols

The training protocols consisted of five exercises, which activated large or small muscle masses and were performed in the following order: bench press, squat, pulldown, overhead press and standing dumbbell curl. All exercises were performed using free weights or universal weight machines. The maximum strength for every exercise was measured using the 1RM method^41^.

The training protocols were designed in conformity with previous studies^1, 5^. In brief, the SE protocol consisted of three sets of 16–20 repetitions at 40% of the 1RM intensity with a 1-minute rest interval between exercises and sets. The MH protocol consisted of three sets of 12 repetitions at 70% of the 1RM intensity with a 2-minute rest interval between exercises and sets. The MS protocol consisted of four sets of 6 repetitions at 90% of the 1RM intensity with a 3-minute rest interval between exercises and sets. All subjects used a complete range of motion and a cadence of a 1- to 2-second positive phase and a 1- to 2-second negative phase. The total workload for the three RT protocols was kept as similar as possible.

### Experimental protocol

The subjects checked into the laboratory at 4 p.m. and did not perform physical exercise for 72 h before the experimental session. Each subject performed the experimental sessions at the same time of day. All subjects performed a 5-minute warm-up of treadmill walking and calisthenics and a specific warm-up for each exercise with a constant range of motion and without an external load; this warm-up consisted of approximately 10 repetitions, and then, the subjects had a 5-minute recovery interval. Each group performed experiments according to their training protocols.

### Blood samples

During each acute exercise experiment, five milliliters of blood was collected before exercise and immediately, 1 h and 24 h after exercise in standard anticoagulant (EDTAK2)-treated vacutainer tubes for every subject. All blood samples were centrifuged at 1500 × g for 10 minutes immediately after each blood draw to pellet cellular elements and then centrifuged at 10,000 × g for 5 minutes at 4 °C to completely remove cell debris. The supernatant plasma was then collected and immediately frozen at −80 °C.

### Biochemical analyses

Blood samples were collected before and immediately and 1 h after exercise for determination of LA. Blood LA was determined using an automatic lactate analyzer (EKF Diagnostic GmbH, Barleben, Germany). Testosterone and cortisol were measured using chemiluminescent microparticle immunoassays (Beckman Coulter Inc., Brea, CA, USA). IGF-1 was measured using chemiluminescence immunoassays (Diagnostic Products Corporation, Los Angeles, USA). IL-6 was measured using electrochemiluminescence immunoassays (Roche Diagnostics, Mannheim, Germany). CK and hs-CRP were measured using an automatic clinical chemistry analyzer (Hitachi 7600, Japan). The concentration of IL-10 was measured using a commercial radioimmunoassay kit (Beijing North Institute of Biological Technology, China).

### Circulating miRNA screening using a TaqMan Low Density Array

The TLDA was used as described previously^42^. For the three RT protocols, an equal volume of plasma from 10 participants was mixed separately to form the Pre and 0 h sample pools (each sample pool contained 10 ml). RNA was isolated from each pooled sample using TRIzol reagent, and reverse transcription was carried out using a TaqMan MicroRNA Reverse Transcription Kit and Megaplex RT Primers. The miRNA screening of 754 different human miRNAs was performed using the TLDA on an ABI PRISM 7900HT Fast Real-Time PCR System (Applied Biosystems). The concentrations of plasma miRNAs were normalized to Let-7d/g/i trio^43^. The results are shown using the Ct (cycle threshold) value and normalized to the calculated mean Ct value of the Let-7d/g/i of each pooled sample (ΔCt). The relative expression was determined using the comparative Ct method (2^−ΔΔCt^).

### RNA isolation and quantification of circulating miRNAs

RNA isolation and RT-qPCR were performed as described previously^42^. The total RNA, including miRNAs, was extracted from 100 µL plasma using a 1-step phenol/chloro form purification protocol. To control for variability in the RNA extraction and purification procedures, all samples from a given subject were handled in the same batch. Hydrolysis probe-based RT-qPCR was carried out using a TaqMan PCR kit and an Applied Biosystems 7300 Sequence Detection System^44^. The Ct values were determined using default threshold settings, and the average Ct value was calculated from triplicate PCRs. Ct values were normalized to the Let-7d/g/i trio, and the fold change of individual miRNA was determined using the 2^−ΔΔCt^ equation. The ΔCt was calculated by subtracting the Ct values of the Let-7d/g/i trio from the mean Ct values of the target miRNAs. The ΔCt values were then compared (ΔΔCt) with each participant’s own resting baseline value at the Pre time point (normalized to a fold change of 1).

### Statistical analysis

The GraphPad Prism 5 and SigmaPlot 10.0 packages were used. Data are presented as the means ± standard error of the mean (SEM). The normality of the data distribution was tested using the Shapiro-Wilk normality test. The non-parametric Friedman test was performed to compare miRNA, inflammatory cytokine and muscle damage marker concentrations. The differences in other variables were compared using repeated measures ANOVA. When appropriate (*p* value < 0.05), a Dunn multiple comparison (miRNAs, inflammatory cytokines and muscle damage markers) or a Bonferroni multiple comparison (other variables) *post hoc* test was used to compare groups of different time points. Each result labeled with *p* < 0.05 indicates *p* values that resulted from the *post hoc* test. Correlations of miRNA profiles between baseline and immediately after exercise were calculated using Pearson correlation analysis, and correlations of miRNAs and other blood parameters were performed using Spearman rank correlation analysis as appropriate for the data distribution. A *p* value < 0.05 was considered statistically significant.

## Electronic supplementary material

Supplementary Information
