# Supplementary material for: Time-course responses of circulating microRNAs to three resistance training protocols in healthy young men
Source: Sci Rep. 2017 May 19;7:2203. doi: 10.1038/s41598-017-02294-y (PMC5438360; doi:10.1038/s41598-017-02294-y)
Supplement: Supplementary file 1 — Supplementary Information [file 41598_2017_2294_MOESM1_ESM.pdf]

Supplementary Information

Time-course responses of circulating microRNAs to three resistance training protocols in healthy young men

Shufang Cui[1], Biao Sun[2], Xin Yin[2], Xia Guo[3], Dingming Chao[3], Chunni Zhang[1,4], Chen-Yu Zhang[1*], Xi Chen[1*]& Jizheng Ma[1,3*]

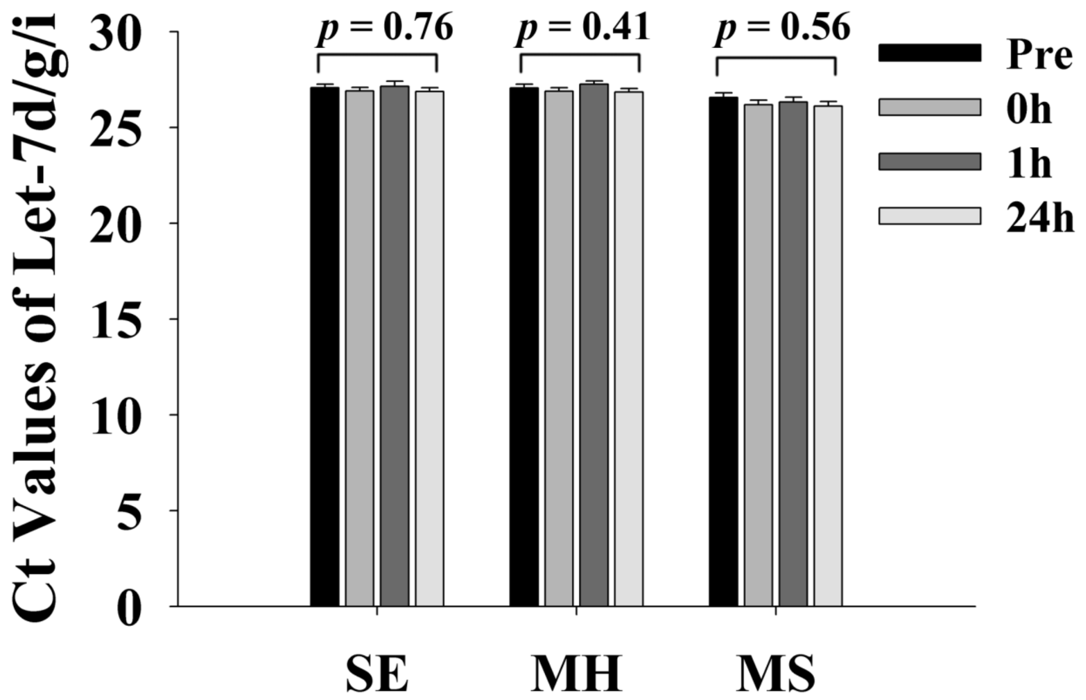

Supplementary Figure S1. The Ct values of Let-7d/g/i in plasma samples for SE, MH and MS groups. The total amount of Let-7d/g/i trio was simultaneously measured in a same RT-qPCR reaction. Let-7d, Let-7g and Let-7i were reverse-transcribed in a single reaction using a mixture of stem-loop primers of Let-7d, Let-7g and Let-7i (in the ratio of 1：1：1). Accordingly, real-time PCR was performed using a TaqMan miRNA probe pool of Let-7d, Let-7g and Let-7i (in the ratio of 1：1：1). Ct values of every individual's Let-7d/g/i before and after each RT protocol almost remained unchanged. For each subject, Ct values of Let-7d/g/i were obtained at baseline (Pre), immediately after exercise (0 h), after1 h of recovery (1 h) and after 24 h of recovery (24 h).

Supplementary Table S1. The validated target genes regulated by miRNAs that were found to be significantly altered in the three different RT conditions.

miRNAs	Validated targets	Biological function	References
miR-133a	DNM2, FGFR1, Foxl2， PP2AC, Prdm16, UCP2	Promotion of myoblast differentiation, regeneration, inhibition of cell proliferation, brown adipose determination, regulation of energy expenditure, thermogenesis, myofiber conversion.	1-5
miR-133b	FGFR1, PP2AC, Prdm16	Promotion of myoblast differentiation, inhibition of cell proliferation, brown adipose determination.	3,4
miR-181a	Hoxa11, PVALB4， Sirt1	Promotion of myoblast differentiation, regulation of myotube size, skeletal muscle relaxation.	6-8
miR-21	Il12a, PDCD4， PTEN	Regulation of cytokine production and immune response in multiple context.	9-14
miR-206	BDNF, Gja1, CCND1, Fstl1, HDAC4, KSRP, Notch3, Pax3, Pax7, Utrn, Vegfa	Promotion of myoblast differentiation, apoptosis, inhibition of myoblast fusion, skeletal-muscle regeneration, control of angiogenesis.	15-25
miR-208b	Sox6, Sp3, Thrap1	Promotion of muscle growth and hypertrophy, Control of skeletal muscle fiber type.	26-28
miR-221	A20, ADIPOR1, CXCL12, IRF2, Myod1, Cdkn1b, Cdkn1c, SOCS1, TOX	Promotion of fully differentiated muscle phenotype, cardiomyocyte hypertrophy, regulation of myogenic microRNA expression, myogenesis, regeneration. Promotion of proinflammatory cytokines production, regulation of endothelial inflammatory response, myocarditis, neuroinflammation.	29-34

Reference

1. Chen, X. *et al.* In vitro evidence suggests that miR-133a-mediated regulation of uncoupling protein 2 (UCP2) is an indispensable step in myogenic differentiation. *J Biol Chem* **284,** 5362-5369 (2009).
2. Liu, N. *et al.* Mice lacking microRNA 133a develop dynamin 2-dependent centronuclear myopathy. *J Clin Invest* **121,** 3258-3268 (2011).

3. Feng, Y. *et al.* A feedback circuit between miR-133 and the ERK1/2 pathway involving an exquisite mechanism for regulating myoblast proliferation and differentiation. *Cell Death Dis* **4,** e934 (2013).
4. Yin, H. *et al.* MicroRNA-133 controls brown adipose determination in skeletal muscle satellite cells by targeting Prdm16. *Cell Metab* **17,** 210-224 (2013).
5. Luo, Y. *et al.* microRNA133a targets Foxl2 and promotes differentiation of C2C12 into myogenic progenitor cells. *DNA Cell Biol* **34,** 29-36 (2015).
6. Soriano-Arroquia, A., House, L., Tregilgas, L., Canty-Laird, E. & Goljanek-Whysall, K. The functional consequences of age-related changes in microRNA expression in skeletal muscle. *Biogerontology* **17,** 641-654 (2016).
7. Naguibneva, I. *et al.* The microRNA miR-181 targets the homeobox protein Hox-A11 during mammalian myoblast differentiation. *Nat Cell Biol* **8,** 278-284 (2006).
8. Chu, W. Y. *et al.* Rapid Muscle Relaxation in Siniperca chuatsi is Coordinated by Parvalbumin (PVALB) and MiR-181a. *Curr Mol Med* **15,** 772-779 (2015).
9. Iliopoulos, D., Jaeger, S. A., Hirsch, H. A., Bulyk, M. L. & Struhl, K. STAT3 activation of miR-21 and miR-181b-1 via PTEN and CYLD are part of the epigenetic switch linking inflammation to cancer. *Mol Cell* **39,** 493-506 (2010).
10. Sheedy, F. J. *et al.* Negative regulation of TLR4 via targeting of the proinflammatory tumor suppressor PDCD4 by the microRNA miR-21. *Nat Immunol* **11,** 141-147 (2010).
11. Ruan, Q. *et al.* The microRNA-21-PDCD4 axis prevents type 1 diabetes by blocking pancreatic beta cell death. *Proc Natl Acad Sci U S A* **108,** 12030-12035 (2011).
12. Merline, R. *et al.* Signaling by the matrix proteoglycan decorin controls inflammation and cancer through PDCD4 and MicroRNA-21. *Sci Signal* **4,** ra75 (2011).
13. Das, A., Ganesh, K., Khanna, S., Sen, C. K. & Roy, S. Engulfment of apoptotic cells by macrophages: a role of microRNA-21 in the resolution of wound inflammation. *J Immunol* **192,** 1120-1129 (2014).
14. Lu, T. X., Munitz, A. & Rothenberg, M. E. MicroRNA-21 is up-regulated in allergic airway inflammation and regulates IL-12p35 expression. *J Immunol* **182,** 4994-5002 (2009).
15. Chen, J. F. *et al.* microRNA-1 and microRNA-206 regulate skeletal muscle satellite cell proliferation and differentiation by repressing Pax7. *J Cell Biol* **190,** 867-879 (2010).
16. Anderson, C., Catoe, H. & Werner, R. MIR-206 regulates connexin43 expression during skeletal muscle development. *Nucleic Acids Res* **34,** 5863-5871 (2006).
17. Hirai, H. *et al.* MyoD regulates apoptosis of myoblasts through microRNA-mediated down-regulation of Pax3. *J Cell Biol* **191,** 347-365 (2010).
18. Gagan, J., Dey, B. K., Layer, R., Yan, Z. & Dutta, A. Notch3 and Mef2c proteins are mutually antagonistic via Mkp1 protein and miR-1/206 microRNAs in differentiating myoblasts. *J Biol Chem* **287,** 40360-40370 (2012).
19. Stahlhut, C., Suarez, Y., Lu, J., Mishima, Y. & Giraldez, A. J. miR-1 and miR-206 regulate angiogenesis by modulating VegfA expression in zebrafish. *Development* **139,** 4356-4364 (2012).

20. Dey, B. K., Gagan, J. & Dutta, A. miR-206 and -486 induce myoblast differentiation by downregulating Pax7. *Mol Cell Biol* **31,** 203-214 (2011).
21. Rosenberg, M. I., Georges, S. A., Asawachaicharn, A., Analau, E. & Tapscott, S. J. MyoD inhibits Fstl1 and Utrn expression by inducing transcription of miR-206. *J Cell Biol* **175,** 77-85 (2006).
22. Dai, Y. *et al.* The role of microRNA-1 and microRNA-206 in the proliferation and differentiation of bovine skeletal muscle satellite cells. *In Vitro Cell Dev Biol Anim* **52,** 27-34 (2016).
23. Amirouche, A. *et al.* Converging pathways involving microRNA-206 and the RNA-binding protein KSRP control post-transcriptionally utrophin A expression in skeletal muscle. *Nucleic Acids Res* **42,** 3982-3997 (2014).
24. Miura, P., Amirouche, A., Clow, C., Belanger, G. & Jasmin, B. J. Brain-derived neurotrophic factor expression is repressed during myogenic differentiation by miR-206. *J Neurochem* **120,** 230-238 (2012).
25. Alteri, A. *et al.* Cyclin D1 is a major target of miR-206 in cell differentiation and transformation. *Cell Cycle* **12,** 3781-3790 (2013).
26. Callis, T. E. *et al.* MicroRNA-208a is a regulator of cardiac hypertrophy and conduction in mice. *J Clin Invest* **119,** 2772-2786 (2009).
27. Kim, J. M. *et al.* A polymorphism in the porcine miR-208b is associated with microRNA biogenesis and expressions of SOX-6 and MYH7 with effects on muscle fibre characteristics and meat quality. *Anim Genet* **46,** 73-77 (2015).
28. van Rooij, E. *et al.* A family of microRNAs encoded by myosin genes governs myosin expression and muscle performance. *Dev Cell* **17,** 662-673 (2009).
29. Cardinali, B. *et al.* Microrna-221 and microrna-222 modulate differentiation and maturation of skeletal muscle cells. *PLoS One* **4,** e7607 (2009).
30. Togliatto, G. *et al.* Unacylated ghrelin promotes skeletal muscle regeneration following hindlimb ischemia via SOD-2-mediated miR-221/222 expression. *J Am Heart Assoc* **2,** e000376 (2013).
31. Tan, S. B. *et al.* Small molecule inhibitor of myogenic microRNAs leads to a discovery of miR-221/222-myoD-myomiRs regulatory pathway. *Chem Biol* **21,** 1265-1270 (2014).
32. Wang, C. *et al.* MiR-221 promotes cardiac hypertrophy in vitro through the modulation of p27 expression. *J Cell Biochem* **113,** 2040-2046 (2012).
33. Corsten, M. *et al.* The microRNA-221/-222 cluster balances the antiviral and inflammatory response in viral myocarditis. *Eur Heart J* **36,** 2909-2919 (2015).
34. Xia, L., Zhang, Y. & Dong, T. Inhibition of MicroRNA-221 Alleviates Neuropathic Pain Through Targeting Suppressor of Cytokine Signaling 1. *J Mol Neurosci* (2016).
